# Selective Impairment of Attentional Networks of Alerting in Wilson's Disease

**DOI:** 10.1371/journal.pone.0100454

**Published:** 2014-06-20

**Authors:** Yongsheng Han, Fangfang Zhang, Yanghua Tian, Panpan Hu, Bo Li, Kai Wang

**Affiliations:** 1 Department of Neurology, The First Affiliated Hospital of Anhui Medical University, Hefei, Anhui Province, PR China; 2 Institute of Neurology, Anhui University of Chinese Medicine, Hefei, Anhui Province, PR China; Centre de Neuroscience Cognitive, France

## Abstract

Wilson's disease (WD) is typically affected by attention, which is one of the cognitive domains. The Attention Network Test (ANT) was developed to measure the functioning of the following three individual attentional networks: orienting, alerting, and executive control. The ANT has been used in a variety of neuropsychiatric conditions; however, it has not been used in WD. The aim of this study was to investigate the attentional function of WD patients, and 35 patients with early and moderate neurological WD, as well as 35 gender-, age-, and education-matched healthy controls performed the ANT. Remarkable differences between the patients and healthy controls were observed in the alerting network (*p* = 0.007) in contrast the differences in the orienting (*p* = 0.729) and executive control (*p* = 0.888) networks of visual attention. The mean reaction time in the ANT was significantly longer in the WD patients than in the controls (*p<*0.001, 0.001). In the WD patients, there was an effect specifically on the alerting domain of the attention network, whereas the orienting and executive control domains were not affected.

## Introduction

Wilson's disease (WD) is a rare autosomal recessive genetic disorder of copper metabolism that is characterized by hepatic and neurological symptoms. In 1912, Kinnier Wilson first described the disease as a syndrome that appears in between one in 30,000 and one in 100,000 individuals [1]. The consequences of this disorder are related to copper deposition in different tissues including the brain, liver, kidneys, and cornea [2]. Different parts of the brain are affected including the brainstem, cerebellum, thalamus, and subcortical white matter, whereas the most common damage frequently occurs in the basal ganglia [3], [4], [5]. The clinical expression is highly variable, with hepatic, neurological, or psychiatric symptoms predominating.

Approximately fifty percent of WD patients have psychiatric or neurological problems [6]. Cognitive changes have been reported since the first WD cases were described in 1912, although dystonia and dysarthria are the most common neurological signs. One of the most common impairments is memory change [7]; other cognitive changes have been reported in untreated cases, including dementia [8]. Neuropsychological evaluation evokes cognitive deficits in numerous domains, including focused attention, verbal learning, visuo-constructive ability, mental speed, verbal fluency, set-shifting ability, and visual memory, which are the most frequently damaged cognitive domains in WD patients [9]. Portala et al. [10] reported that symptomatic WD patients showed remarkably lower performance than normal controls on assessments of finger tapping, simple reaction time, short-term memory, word decoding speed, grammatical reasoning and maze perception.

Attention deficit is one of the most frequent symptoms of cognitive impairment in WD patients. In various studies, WD patients have been reported to have a deficit in one of the attentional systems. Seniow et al. observed marked attention impairment in a digit span test in WD patients [11]. Portala et al. found that 62% of WD patients present concentration difficulties and executive control problems in the allocation of attention [10], [12]. Hegde et al. [4] reported that nearly one-half of WD patients experience impairment in sustained and focused attention. Lin et al. [13] reported on a child with WD with an attention deficit who had been misdiagnosed with attention deficit hyperactivity disorder (ADHD) for more than a year because he could not pay close attention and frequently left his seat in the classroom.

WD, Parkinson's disease (PD) and Huntington's disease (HD) are basal ganglia disorders. Zhou et al. [14] found that in using the attentional network test (ANT) to test the attention network, PD patients showed selective abnormality in the orienting network, and the alerting and executive control networks were not significantly different between the PD patients and the healthy controls. Couettea and his coworker reported attention deficit in HD patients, with a slowing of disengagement processes, which delayed the occurrence of exogenous processes such as inhibition of return (IOR) as well as endogenous strategies and re-orienting towards uncued locations where the testing target was expected to appear [15].

These studies show that PD and HD patients have impaired attention. Additionally, WD is a basal ganglia disorder, and we hypothesized that individuals with WD would have attention network function difficulties, with the basal ganglia playing a key role in impaired attention in WD patients. Differing from patients with other basal ganglia disorders, WD patients have obvious and visible MRI changes that provide a unique opportunity for exploration of attentional cognitive deficits in the substrate, leading to a better understanding of the role of the basal ganglia structures in higher cognitive functions [9]. The anatomy and connections of the basal ganglia indicate that these structures are crucial links between the parts of the brain which have classically been considered to be related to emotional functioning and that brain regions formerly considered to have largely motor functions. The basal ganglia play a very important role in the development and integration of psychomotor behaviors such as motor functioning, memory and attention [16].

On the basis of numerous neuroanatomical and cognition studies, Posner and Petersen divided the human attention system into three independent networks identified as alerting, orienting and executive control. Alerting is defined as the capability of completing and maintaining response readiness for an impending stimulus. Orienting is the selection of information from multiple sensory inputs. Executive control describes the ability to work out a conflict when faced with competing responses [17]. Each network is connected to specific anatomical areas and is dependent on specific neuromodulators [17]. The thalamic, frontal and parietal regions of the right hemisphere have links with the alerting system, and norepinephrine from the midbrain nucleus coeruleus regulates the alerting system. Sections of the superior parietal lobe, the temporoparietal junction and the frontal eye fields appear to be activated by the orienting system, which is modulated by acetylcholine. The midline frontal areas (the anterior cingulated cortex) and lateral prefrontal cortical regions are activated by the executive control network, which is modulated by dopamine [17], [18].

The attentional network test (ANT) was developed to evaluate the efficiencies of the three attention networks in one experiment and was designed for normal subjects and various patient populations [17]. It has detected dissociations in the different networks in various populations [14], [19], [20], [21], [22]. No study has been published on the attention networks of WD patients. Attention is a vital prior condition for every ‘‘higher-order’’ cognitive process, such as working memory and executive function [23], [24], [25], ensuring that attention is very important. Whether there is a global attention deficit or a deficit in a specific attention network in WD patients is unknown. The primary goal of this study was to investigate whether specific changes in attentional networks could be found in WD patients versus healthy controls (HC) matched for age, gender, and educational level. We attempted to access the different attentional subcomponents and determine which were impaired or unaffected in the identical patient group. We hypothesized that WD patients would be more impaired than the HCs in attention tasks; however, this impairment depends on the attentional subcomponent involved in the task.

## Materials and Methods

### Ethics Statement

The study protocol was approved by the ethics committee of the Anhui Medical University. Each participant provided signed informed consent, and this research was in accordance with the Helsinki Declaration.

### Subjects

We studied 35 WD patients and 35 HCs. The patients were recruited from the Institute of Neurology at Anhui University of Chinese Medicine, China. The WD diagnosis criteria [26] included the following: (1) presentation of extrapyramidal symptoms and signs, (2) corneal Kayser-Fleischer rings observed with a slit lamp, (3) serum ceruloplasmin <20 mg/dL or copper oxidase <0.21 mg/dL, and (4) a 24-h urinary copper concentration >100 *µ*g. Cranial magnetic resonance imaging (MRI) or computerized tomography (CT) scans were obtained for all the patients (thirty-four WD patients had MRI scans whereas one WD patient had a CT scan because sheet metal placement in his body prohibited an MRI scan). The following exclusion criteria included (1) patients with mental retardation (a score on the Wechsler Adult Intelligence Scale-Revised Chinese Version [WAIS-RC]-IQ <70 points), (2) patients with dysaudia and lalopathy, (3) patients with significant impairment of liver function (alanine aminotransferase >100 U or patients with liver cirrhosis), (4) patients with possible anxiety and depression (Hamilton Anxiety Scale [HAMA] and Hamilton Depression Scale [HAMD] >7 points), and (5) patients taking L-dopa or other drugs that affect cognitive function. No patients had visual acuity or field deficits. The patients were evaluated using the United Wilson's Disease Rating Scale (UWDRS) [27] to assess the entire spectrum of clinical symptoms. All the patients were receiving regular copper chelation therapy.

Thirty-five HCs matched for age, gender, education level, and intellectual level were recruited from a local volunteer group. All the subjects were right-handed, with normal speaking, writing, language expression, visual acuity, and comprehension skills. None of the HCs had a history of serious physical or mental illness. The clinical and demographic data of the WD patients and the HCs are shown in Table 1.

**Table 1:** Clinical and demographic data and Neuropsychological task performances of WD patients and HCs.

		WD (*N* = 35)	HC (*N* = 35)	*P*
Gender (M:F)		23/12	24/11	-
Age at investigation (years,mean±*SD*)		22.66 (3.50)	22.80 (3.42)	0.863
Years of school education (years,mean±*SD*)		11.26 (2.76)	11.60 (3.13)	0.629
Wechsler Adult Intelligence Scale-Revised Chinese version (WAIS-RC) (mean±*SD*)		99.34 (10.84)	101.53 (11.98)	0.388
Verbal fluency (mean±*SD*)		11.20 (2.15)	10.80 (1.48)	0.362
DT	Digits forwards (mean±*SD*)	7.77 (0.55)	7.94 (0.34)	0.119
	Digits backwards (mean±*SD*)	4.97 (1.15)	5.06 (1.18)	0.547
Hamilton Anxiety Scale (HAMA) (mean±*SD*)		2.86 (2.05)	2.14 (2.20)	0.164
Hamilton Depression Scale (HAMD) (mean±*SD*)		2.94 (2.09)	2.25 (2.20)	0.185
United Wilson's Disease Rating Scale (UWDRS) (mean±*SD*)		19.43 (9.86)	-	-
Duration of disease (years,mean±*SD*)		6.68 (4.94)	-	-

### Neuropsychological background tests

The neuropsychological tests performed with all the subjects, comparing the HCs with the WD patients were as follows: (1) the WAIS-RC [28] was performed to measure intelligence; (2) the HAMA [29] and HAMD [30] were used to measure anxiety and depressive states; (3) a verbal fluency (animals/min) test [31], [32] was applied to measure the frontal lobe functions; and (4) the digit span test [33], including forward and backward digits, investigated short-term memory and attention span.

### Attention network test

**Figure 1:**
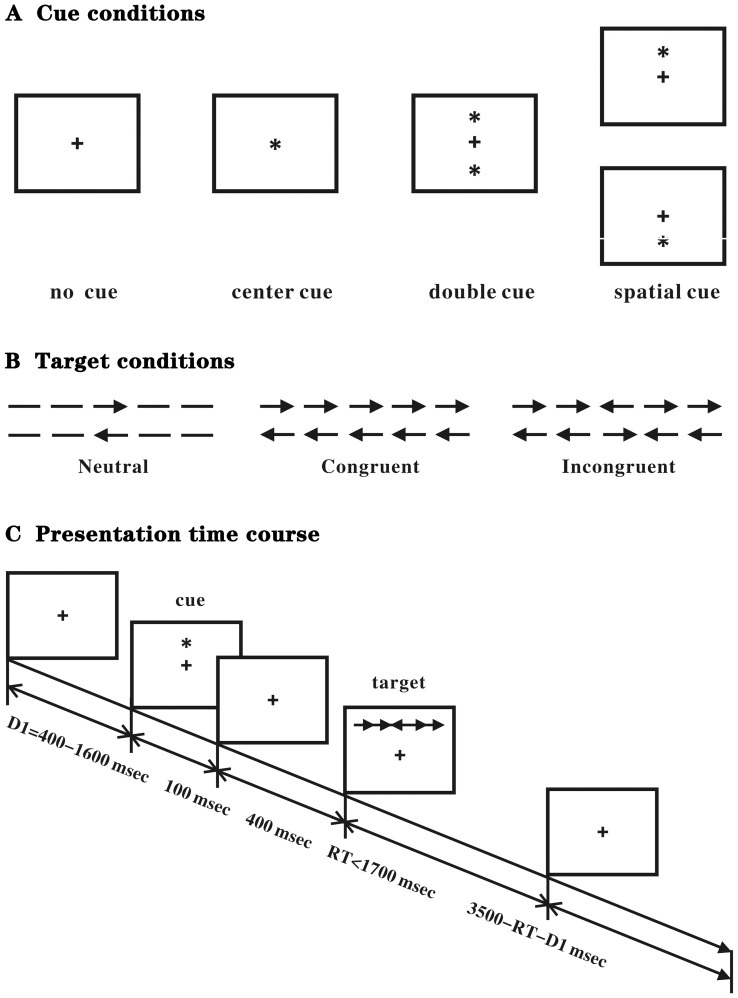
Experimental paradigm of the Attention Network Test. (A) The 4 cue conditions. (B) The 6 stimuli used in the present experiment. (C) An example of the procedure.

The ANT was used as described by the authors of the test [17] to assess the attentional networks of the participants. A computerized 30-min task is designed to bond cued reaction time (RT) with a flanker task. The participants observed the stimuli shown on the computer screen and responded by pressing two response buttons. The stimuli comprised a row of five visual horizontal black lines that have arrowheads which point left or right, against a gray background. Whether the sides of two arrows are in the same, opposite or no direction determine the congruent, incongruent or neutral condition. The participants were required to decide the direction of a central arrow and press a left or a right key in accordance with the pointing direction. A series of spatial and alerting cues and flankers were introduced to test all three attentional networks, as described. The participants were guided to concentrate on a central cross-shaped fixation point presented for randomized 400 to 1600 ms, which was subsequently replaced for 100 ms by one of four warning cues that provide information about the impending target. The central arrow as a target could appear above or below the fixation point and was in the middle of two flankers (arrows). Different equal-balanced-cues and flankers appeared in random order. The participants accomplished a 24-trial practice block and feedback was included after each trial. The subjects completed three experimental blocks of 96 trials with no feedback, with up to 2 min of rest between the blocks. The reaction times (RT) and accuracy were recorded. The four cue conditions were as follows: (1) no cue, the subjects were shown a cross for 100 ms that was the same as the first fixation; (2) a central cue, which was at the central fixation point; (3) a double cue, in which the cues were presented on the two possible target locations simultaneously (above and below the fixation point); and (4) a spatial cue, in which the cue was presented on the target location (above or below the central fixation point). The trials were presented randomly. Throughout the task, the participants were guided to concentrate on a centrally located fixation cross and to respond as rapidly and as accurately as possible (see Figure 1).

### Calculation of attention network efficiencies

The differences in RTs originated from various experimental conditions to measure the alerting, orienting and executive control networks [17]. From the following raw reaction time data, the attention network efficiency values were calculated. The raw RTs with correct responses were omitted with a 100–1700 ms window to eliminate the effect from the outliers, and the RTs outside this window were not included. Subtracting the mean of the median RTs of the conditions with a double cue from the mean of the median RTs of the conditions with no cue could yield the alerting effect. Because neither of these conditions provides information on the spatial location of the target, subtraction provides the measure of alerting. The orienting effect was calculated by subtracting the mean of the median RTs of the conditions with a spatial cue from the mean of the median RTs of the conditions with a center cue. The subject was alerted in both conditions, however, only the spatial cue offered spatial information on where to orient. The conflict (executive) effect was calculated by subtracting the mean of the median RTs of the conditions with the congruent flankers from the mean of the median RTs in the conditions with the incongruent flankers.

### Statistical analysis

SPSS 13.0 software was used in all the analyses. For the two-tailed tests, the level of significance was set at *p<0.05*. The group differences were evaluated by using nonparametric tests for 2-independent samples (Mann-Whitney U-test). The relationship between the patients' performance in the ANT and their clinical or neuropsychological ability was computed using Spearman's correlation.

## Results

### The neuropsychological background data

**Figure 2:**
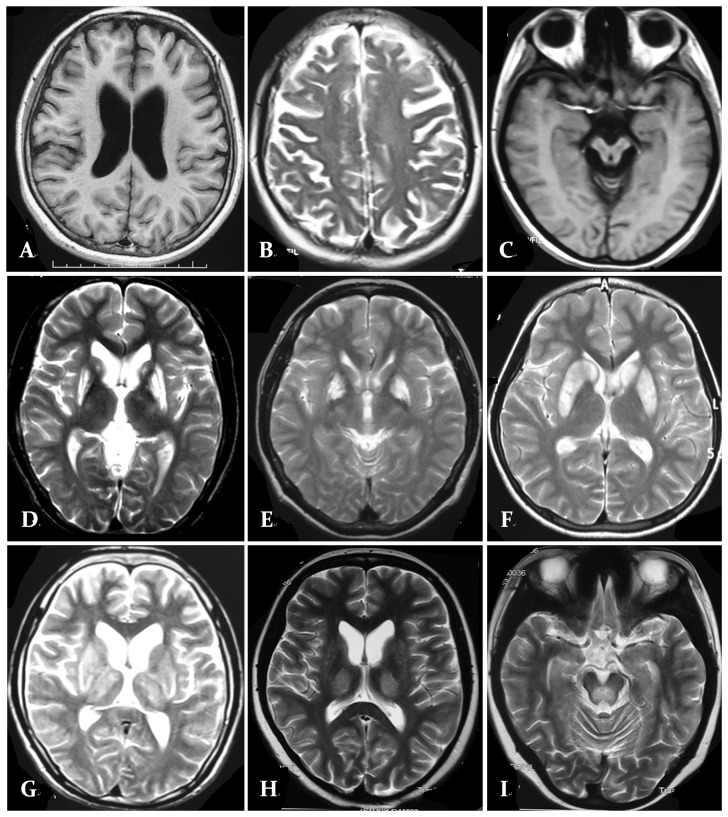
MRI with classically described changes in patients with Wilson '**s disease.** **A.** Axial T1-W imaging a 18-year-old male patient shows diffuse subcortical atrophy. **B.** Axial T2-W imaging a 22-year-old male patient shows diffuse cortical atrophy. **C.** Axial T1-W imaging a 22-year-old male patient shows brainstem atrophy. **D.** Axial T2-W imaging a 24-year-old female patient shows bilateral putamen hyperintensity signal abnormalities. **E.** Axial T2-W imaging a 25-year-old male patient shows bilateral globus pallidus hyperintensity signal abnormalities. **F.** Axial T2-W imaging a 23-year-old male patient shows bilateral putamen and caudate nucleus hyperintensity signal abnormalities. **G.** Axial T2-W imaging a 20-year-old male patient shows bilateral basal ganglionic and thalamic hyperintensity in addition to diffuse atrophy . **H.** Axial T2-W imaging a 26-year-old female patient shows bilateral thalamic hyperintensity signal abnormalities . **I.** Axial T2-W imaging a 21-year-old male patient shows pons hyperintensity signal abnormalities.

The means and standard deviations of the demographic and clinical characteristics of the WD patients and HCs showed no remarkable differences in gender, age and education; the WAIS-RC, HAMA, HAMD, DT, and VFT scores of the two groups are shown in Table 1. Thirty-four patients underwent MRI examinations, and the data showed instances of basal ganglia abnormality (18 of 34 had a high T2 signal and/or a lower T1 signal on MRI), mild cortical and/or subcortical atrophy (13 of 34), brainstem abnormality (10 out of 34) and thalamus abnormality (11 of 34); one patient had a CT scan that showed a mild subcortical atrophy (Figure 2).

### The efficiencies of the three networks

**Figure 3:**
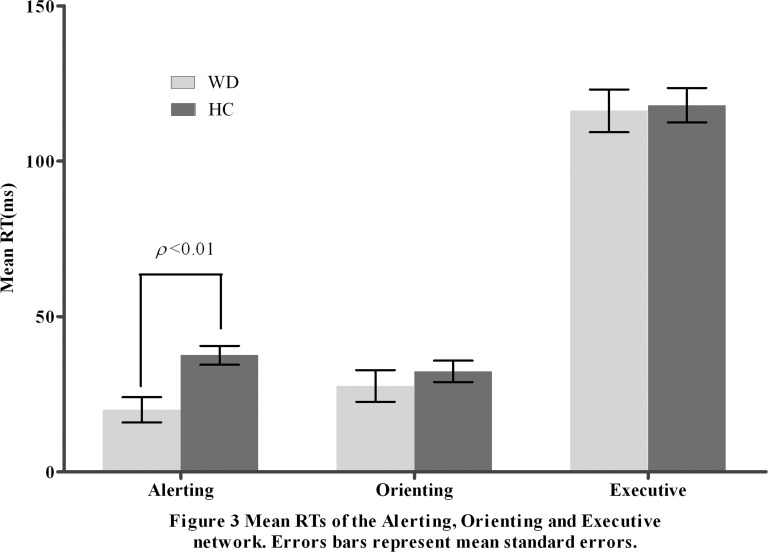
Mean RTs of the Alerting, Orienting and Executive network. Errors bars represent mean standard errors.

Table 2 shows the mean raw RTs and accuracy for each cue condition of the ANT in the two groups. Table 3 and Figure 3 summarize the mean score and the standard error *(SE)* for each of the attention networks, the mean RT, and the global accuracy. There were longer (*Z* = −4.305, *p*<0.001) overall mean RTs in the WD patients than in the HCs; the accuracy rates were similar (*Z* = −1.689, *p*  = 0.091) in the two groups. Between the WD patients and the HCs, we found significant differences (*Z* = −2.721, *p* = 0.007) in the baseline RT-adjusted alerting network scores. The differences between the groups in the orienting and executive network scores were insignificant (*Z* = −0.347, *p* = 0.729; *Z* = −0.141, *p* = 0.888).

**Table 2:** Mean reaction times and accuracy under each cue condition for WD patients and HCs.

Group	Flanker	Cue type
		No cue	Double cue	Center cue	Spatial cue
Mean RTs (ms) and standard deviations					
WD	Congruent	720 (71)	692 (84)	705 (75)	668 (70)
	Incongruent	824 (67)	812 (77)	822 (71)	789 (76)
	neutral	656 (80)	625 (77)	629 (84)	610 (79)
Health Controls	Congruent	645 (97)	600 (95)	618 (95)	585 (102)
	Incongruent	752 (96)	732 (95)	742 (94)	702 (100)
	neutral	582 (93)	524 (90)	538 (93)	512 (92)
Accuracy (%) and standard deviations					
WD	Congruent	0.99 (0.02)	0.99 (0.02)	0.99 (0.03)	0.99 (0.02)
	Incongruent	0.96 (0.06)	0.95 (0.06)	0.97 (0.03)	0.96 (0.06)
	neutral	0.99 (0.02)	0.98 (0.04)	0.99 (0.02)	0.99 (0.03)
Health Controls	Congruent	0.99 (0.06)	0.99 (0.06)	0.99 (0.06)	0.99 (0.06)
	Incongruent	0.96 (0.09)	0.99 (0.08)	0.96 (0.08)	0.97 (0.09)
	neutral	0.99 (0.06)	0.96 (0.08)	0.99 (0.06)	0.99 (0.06)

**Table 3:** Attention network scores (in RT and ratio score) and accuracy (%) of WD patients and HCs.

	WD (*N* = 35) Mean±*SE*	HC (*N* = 35)Mean±*SE*	*Z*	*P*
Alerting RT (ms)	20.00 (4.10)	37.54 (3.06)	−2.721	0.007
Ratio	0.029 (0.006)	0.061 (0.005)	−3.365	0.001
Orienting RT (ms)	27.66 (5.12)	32.34 (3.45)	−.347	0.729
Ratio	0.039 (0.007)	0.053 (0.006)	−1.204	0.229
Executive RT (ms)	116.20 (6.84)	117.99 (5.55)	−.141	0.888
Ratio	0.166 (0.010)	0.195 (0.014)	−1.380	0.168
Accuracy (%)	97.54 (0.41)	97.49 (1.05)	−1.689	0.091
Mean RT (ms)	709.14 (11.52)	625.80 (15.30)	−4.305	<0.001

The ratio could be applied to verify specific effects that are not affected by the overall reaction time because the response times (RTs) are longer in the WD patients. The median RT in each condition was divided by the participant's overall RT for every subject [20]. The ratio scores are shown in Table 2b. Based on these ratio scores, the WD patients were notably different in regard to the alerting network compared with the controls (*Z = *−3.365, *p = *0.001); there was no significant difference between the orienting and executive networks in the two groups (*Z* = −1.204, *p* = 0.229; *Z* = −1.380, *p* = 0.168).

### Correlations

The examination of the correlations between the networks indicates that the scores for the networks were not significantly related in the WD patients and HCs. We inspected the connection of the scores for the ANT with age and education level as well as the scores on the WAIS-RC, VFT, and UWDRS, and there were no significant interactions among these variables.

## Discussion

The ANT is very sensitive to attention deficits in many neurological and psychiatric disorders such as PD, ADHD, multiple sclerosis (MS), Alzheimer's disease (AD), and schizophrenia [14], [19], [20], [21], [34], [35]. The ANT has the advantage of comparing deficits in different attentional networks [17].

In this study, we first investigated attentional networks based on the ANT in WD patients. The most significant finding is a specific alteration of the alerting network in WD patients. This test is used as indicated by the significantly lower alerting effect in the ANT; no difference between the WD patients and HCs relating to the orienting and the executive control effects was shown. The findings of this study confirm our hypothesis. The deficits of the alerting network indicate that WD patients do not derive as much benefit as the HCs from temporal cue warnings. In accordance with the outcomes of the WAIS-RC, all of the WD patients were not cognitively impaired compared with the HCs, and the lower alerting effect demonstrated in patients suggests a smaller RT difference between the trials that with and without a warning cue. This finding might forecast the harm in using the warning cue to speed up response times or the capability of maintaining alertness with no cue. Even in the trials with no warning cue, the HCs had a more rapid mean RT than the in-trial WD patients with a warning cue (659 ms vs. 719 ms); it is hypothesized that the WD patients with slower overall RTs are lack the ability to completely use the additional helpful information of the warning cue to enhance their reaction times in the cued trials.

The deficit found in WD in alerting attention is not distinct. A similar deficit was found in MS and ADHD, and it was reported in a study that MS and ADHD patients displayed a deficit in the alerting attention network and not in the orienting and executive attention networks [34], [35], [36]. Whether an identical underlying mechanism is involved requires further study.

Despite the existence of three independent attentional networks, some studies have found interactions in test performance, which has been found in HCs and patients [17], [34], [35]. In our study, we did not find any interaction between the alerting, orienting, and executive control networks in WD patients.

Depression, anxiety and tremor are common symptoms of WD, which could impede neuropsychological test performance [37], [38]. In this study, UWDRS evaluation was conducted for WD patients, and they had a neurological UWDRS subscore of under 15. The statistical analysis was impaired by the low prevalence and lower degree of resting tremor and postural tremor in the upper limbs of the WD patients. Although WD movement disorder is not markedly obvious in WD patients, the RTs of the patients with WD are significantly longer than those of the HCs, which contributed to the WD patients having various degrees of upper limb movement awkwardness or tremor. The ratio score transformations are effective in uncovering the group effects in the processes, in a manner that is independent of global slowing. We calculated a ratio score transformation for each participant by dividing the mean RT and determined each condition by the participant's overall RT (Table 2b) for each of the three attentional networks [14]. In this study, the alerting ratio and the alerting RT of the WD patients were significantly lower than those of the HCs. The motor speed factor is not conducive to less efficient alerting attention in WD patients. Marina found that the most often diagnosed symptoms in WD patients are depression (36%) and anxiety (62%) [39]. In this study, the patients were not different from the controls in anxiety and depression, at least as measured by the HAMA and HAMD. Depression and anxiety do not account for our results. Our patients might be expected not to be especially anxious or depressed because our inclusion criteria asked for the total UWDRS scores to be below 30 in the WD patients and for the participants to have a low degree of physical disability, which might explain the low incidence of anxiety and depression in these patients.

The alerting network is a significant source of attention, and it maintains an adequate level of alertness. For optimal performance, it is crucial [40], [41]. In an fMRI study that uses the ANT, the alerting contrast indicated a strong thalamic involvement as well as activation of the anterior and cortical site [18]. The alerting network manifested a specific decrease in the theta-, alpha-, and beta-band activity in the brain oscillations 200–450 ms after the warning signal [42]. In this study, cerebral MRI showed that approximately 11 of the 35 WD patients had abnormally high thalamus T2 signals and/or lower T1 signals, and 13 of 35 WD patients had cortical and/or subcortical atrophy. When analyzing the performance of the ANT in a study of healthy volunteers, only the alerting network was related to strong activation of the thalamus in the three attentional networks [18]. For decades, the thalamus has been reported to be related to arousal, attention and alertness [43]. In this study, approximately one-third of the patients had abnormal magnetic resonance signals in the thalamus; in most of the WD patients, the abnormal imaging involved the basal ganglia, brainstem signal changes and brain atrophy. Imaging studies examining brain areas have demonstrated that an activation of the thalamic, frontal, and parietal areas, particularly of the right hemisphere are involved in alerting control. In this study, few WD patients had abnormal images in the frontal and parietal areas, and it could be hypothesized that the link between the basal ganglia and cortex is involved in the efficiency of the alerting network. Middleton reported that several areas of the prefrontal cortex involved in higher order cognitive function are targets of output from the basal ganglia [44], [45]. Excessive copper accumulates in the basal ganglia in WD patients. The function of the frontostriatal networks, which are involved in motor and cognitive function, are affected by these changes [46]. This area of investigation requires further study.

The attentional networks have been associated with various neuromodulators [47]. Pharmacological studies indicate that noradrenergic antagonists block the warning effect, whereas is does not influence orienting [48]. In such cases, there appears to be a double dissociation, with norepinephrine (NE) involved predominantly in the alerting network [48]. The decrease in the magnitude of the alerting effect results from the reduced levels of noradrenalin in the brain [48]. PET evidence of nigrostriatal dopaminergic dysfunction has been found in WD patients even after many years of penicillamine treatment. [49]. As shown for AD, the possible contribution of alerted NE levels to changes in the alerting effect in WD should be clarified.

Patients with WD are very vulnerable to the effects of attention, which might influence many other cognitive processes or motor functions; attention could be an important candidate for therapy. Recent studies have attempted to rehabilitate specific attentional networks. Strum et al. [50] found that a computerized rehabilitation program could improve specific attentional networks. Vigilance and alertness are the most elementary functions in the hierarchy, which is valuable for the normal operation of selective attention and divided attention. Allcock et al. reported that slowed RTs in computerized tasks are linked with increased fall frequency in PD patients [51]. Attention training might enhance walking and prevent falls in patients. Further study is required to determine whether attention rehabilitation programs could improve attention and motor function in WD patients.

## Conclusions

Our study suggests that the ANT could be completed in WD patients and could facilitate the exploration of specific disease-related alterations in the alerting network. Future research is merited on the future activation of attentional networks in WD by simultaneous fMRI examination during the performance of ANT by a WD. Future studies should examine the interaction of attentional networks and the neuroendocrine system in WD.
